# A 28-Day Toxicity Study of Tenofovir Alafenamide Hemifumarate by Subcutaneous Infusion in Rats and Dogs

**DOI:** 10.1128/spectrum.00339-21

**Published:** 2021-06-30

**Authors:** Doris Zane, Shane Roller, Josephine Shelton, Roshni Singh, Rachna Jain, Yan Wang, Bing Yang, Melanie Felx, Thomas Alessi, Paul L. Feldman

**Affiliations:** a Intarcia Therapeutics, Inc., Hayward, California, USA; b Intarcia Therapeutics, Inc., Research Triangle Park, California, USA; c Charles River Laboratories, Senneville, Quebec, Canada; University of Sussex

**Keywords:** tenofovir alafenamide, subcutaneous, human immunodeficiency virus, antiretroviral, preexposure prophylaxis

## Abstract

The toxicity of tenofovir alafenamide (TAF) hemifumarate (HF) was evaluated when administered by continuous subcutaneous (s.c.) infusion via an external infusion pump for 28 days to rats and dogs. The toxicokinetics of TAF and two metabolites, tenofovir (TFV) and tenofovir diphosphate (TFV-DP) were also evaluated. After administration of TAF HF in rats and dogs, primary systemic findings supported an inflammatory response that was considered minimal to mild. Gross pathology and histopathologic evaluation of tissue surrounding the s.c. infusion site revealed signs of inflammation, including edema, mass formation, fibrosis, and mononuclear cell inflammation in groups receiving ≥300 μg/kg/day in rats and ≥25 μg/day in dogs. Although these changes were observed in animals receiving vehicle, the severity was greater in animals receiving TAF HF. Changes in the local tissue were considered a TAF HF-mediated exacerbation of an inflammatory response to the presence of the catheter. In rats, systemic and local findings were considered not adverse due to their low severity and reversibility; therefore, the “no observed adverse effect level” (NOAEL) was set at 1,000 μg/kg/day. Because none of the systemic findings were related to systemic exposure to TAF, the systemic NOAEL was set at 250 μg/kg/day in dogs. Due to the severity of the observations noted, a NOAEL for local toxicity could not be established. Although these results might allow for exploration of tolerability and pharmacokinetics of s.c. administered TAF HF in humans, data suggest a local reaction may develop in humans at doses below a clinically relevant dose.

**IMPORTANCE** Human immunodeficiency virus (HIV) infection continues to be a serious global human health issue, with ∼38 million people living with HIV worldwide at the end of 2019. HIV preexposure prophylaxis (PrEP) has introduced the use of antiretroviral therapies as another helpful tool for slowing the spread of HIV worldwide. One possible solution to the problem of inconsistent access and poor adherence to HIV PrEP therapies is the development of subcutaneous (s.c.) depots or s.c. implantable devices that continuously administer protective levels of an HIV PrEP therapy for weeks, months, or even years at a time. We evaluate here the toxicity of tenofovir alafenamide, a potent inhibitor or HIV replication, after continuous s.c. infusion in rats and dogs for HIV PrEP.

## INTRODUCTION

Since its emergence nearly 40 years ago, human immunodeficiency virus (HIV) infection continues to be a serious global human health issue, with an estimated 38 million people living with HIV worldwide at the end of 2019 (1). Decades of global initiatives that have educated the public on how to prevent the spread of HIV and broadened access to rapid diagnostic testing and antiretroviral therapies (ART) have dramatically decreased the number of HIV-related deaths and new infections that are reported annually (2); however, in certain populations, such as men who have sex with men, sex workers and their clients, transgender people, and adolescent girls and young women in southern Africa, significant cultural and socioeconomic barriers prevent access to treatment and/or deter the use of prophylactic measures. As a result, the numbers of new infections in these populations remain higher than in the rest of the world (1, 2).

In the last decade, the approval of Truvada (2012, tenofovir disoproxil fumarate 300 mg/emtricitabine 200 mg) and Descovy (2019, tenofovir alafenamide 25 mg/emtricitabine 200 mg) for HIV preexposure prophylaxis (PrEP) has introduced the use of ART for HIV PrEP as another helpful tool for slowing the spread of HIV worldwide. Unfortunately, the effectiveness of these once-daily oral therapies relies on high patient adherence and regular, consistent access to medical facilities, which remain major impediments in some HIV populations, especially in the developing world, where the burden of HIV is the heaviest (1, 2). One possible solution to the problem of inconsistent access and poor adherence to HIV PrEP therapies is the development of subcutaneous (s.c.) depots or s.c. implantable devices that continuously administer protective levels of an HIV PrEP therapy for weeks, months, or even years at a time. Such a therapy would significantly reduce the frequency that patients would need to visit medical facilities, thereby reducing the access burden and increasing adherence.

Tenofovir alafenamide (TAF) is a prodrug form of tenofovir (TFV) (3), which is a potent inhibitor of HIV replication (4). TAF is selectively converted to TFV in the peripheral blood mononuclear cells (PMBCs) via hydrolysis by cathepsin A (5). TFV is then phosphorylated to form the pharmacologically active anabolite, tenofovir diphosphate (TFV-DP), that inhibits HIV reverse transcriptase and prevents the virus from replicating (6). Because the conversion of TAF to TFV is a selective process, administration of a low dose of TAF results in a relatively high accumulation of TFV-DP in PBMCs (3). This high *in vivo* potency of TAF has made it one of the primary candidates for developing a long-acting, continuous release HIV PrEP therapy. Indeed, several groups have demonstrated the potential utility of continuous release TAF in nonclinical pharmacokinetic models using subdermal implants (7–9) that release pharmacologically relevant levels of TAF for several weeks.

Given the impact that a continuous release form of TAF could have on the HIV epidemic, Intarcia Therapeutics began developing a formulation of TAF that could be used with the Medici Drug Delivery System, which includes a small, implantable osmotic minipump that is capable of delivering potent molecules for up to 1 year in a controlled, predictable manner (10). The safety and reliability of the Medici Drug Delivery System has been demonstrated with the continuous s.c. delivery of exenatide for up to 6 months in animals and humans (11–13).

Although TAF has been proven to be safe when administered orally as the hemifumarate (HF) salt (3) and TFV has been safely administered vaginally, both as a gel (14, 15) and as an intravaginal ring (16, 17), when TAF HF is administered continuously via an s.c. implant, some studies have suggested that a local irritation can develop in the s.c. tissue that surrounds the area of a TAF HF-containing implant (18). Due to a lack of placebo controls or rigorous histopathological evaluation, investigators have been unable to conclusively determine whether or not the observed irritation is a direct reaction to TAF HF or a general reaction to the implants or formulations used.

In order to address the potential for TAF to cause local irritation and inflammation when administered s.c., we have assessed the safety and tolerability of TAF HF in rats and dogs when given as a continuous s.c. infusion. We selected s.c. infusion as the route of administration in order to mimic the intended clinical route of administration while avoiding the complexity of developing a formulation that would be suitable for the Medici Drug Delivery System. Both the rat and dog toxicology studies included a 28-day dosing period, followed by a 28-day recovery period. The objectives of these studies were (i) to determine the potential systemic toxicity of TAF in rats and dogs when administered as the HF salt by continuous s.c. infusion for 28 days, (ii) to rigorously evaluate local irritation and toxicity caused by the administration of TAF HF in the tissue surrounding the infusion site, and (iii) to provide full toxicokinetic evaluation of TAF, TFV, and TFV-DP.

The selection of doses for this study was based primarily on two sources. First, previously reported toxicity data after oral administration of TAF HF to nonclinical species were used to define the relationship between systemic exposure and toxicity (19). Second, a pilot pharmacokinetic and tolerability study in rats and dogs was conducted to understand the dose-exposure relationship of TAF, TFV, and TFV-DP after continuous s.c. administration of TAF HF (unpublished data).

The toxicology of TAF HF after oral administration has been studied extensively in mice, rats, beagle dogs, and monkeys (19). The systemic exposure at the highest dose level chosen for this study was expected to remain below the TFV exposures measured in plasma at the no observed adverse effect level (NOAEL) in rats after 28 days of daily oral administration of TAF HF (NOAEL, 6.25 mg/kg/day) (19). Historically, systemic exposure margins in rats have been based on TFV exposure rather than TAF exposure because of the rapid conversion of TAF to TFV in rat plasma and whole blood, which results in the inability to reliably measure TAF in rat plasma. At the NOAEL of 6.25 mg/kg/day after 28 days of oral administration, findings in rats included reduced body weight, reduced food consumption, changes in hematology and clinical chemistry parameters, dose-related decreases in 1,25(OH)_2_D_3_ (vitamin D), decrease in metaphyseal bone mineral density and bone mineral content, gross and microscopic changes in the thymus and kidney, and atrophy of the cancellous bone in the femur. Mechanistic toxicity studies suggest that TAF might directly inhibit vitamin D production, which results in decreased gastrointestinal absorption of calcium and phosphate and decreased renal reabsorption of calcium (19). In dogs, the daily systemic exposure at the highest dose level chosen for this s.c. study was expected to approximate the known NOAEL in beagle dogs after 9 months of daily oral administration of TAF HF (NOAEL, 2 mg/kg/day) (19). At daily oral doses 3-fold higher than the reported NOAEL from oral administration, findings in dogs included body weight loss, reduction in food consumption, effects on hematology and clinical chemistry parameters, minimal renal toxicity, slightly prolonged PR intervals, dark brown discoloration of the fur (mainly on the extremities), pulmonary findings, macroscopic changes in the kidney, histopathological alterations noted in the kidneys, eyes, lung, spleen, liver, and adrenal glands, decrease in vitamin D, and minimal bone loss.

In a previous 14-day continuous s.c. infusion study in rats in which doses of 0 (vehicle control), 1,080, and 3,600 μg/kg/day of TAF were administered as TAF HF at a rate of 0.150 ml/kg/day, TAF-related edema was observed at the infusion site and demonstrated a time- and dose-dependent increase in incidence and severity (unpublished data). Based on the data from the 14-day tolerability study in rats, the highest dose level chosen for this rat toxicity study (1,000 μg/kg/day) was expected to be well tolerated and achieve measurable and clinically relevant systemic exposure of TFV in rat plasma. The systemic exposures of TFV in the high-dose group of this toxicity study were expected to be less than or comparable to the exposures achieved at the NOAEL in the 28-day toxicity study in rats after daily oral administration of TAF (19). As a result, no changes in body weight, food intake, or mortality were expected at the highest dose level chosen for this study.

In a previous 28-day continuous s.c. infusion study in dogs, TAF HF was administered at doses of 25 and 83.3 μg/kg/day (TAF free base equivalents; *n* = 4 dogs/group [unpublished data]). Compared to vehicle controls, a dose-related increase in incidence and severity of mixed leukocyte infiltration and fibrosis was observed in the tissue surrounding the infusion site. In addition, continuous s.c. infusion of TAF HF at a dose of 83.3 μg/kg/day (TAF free base equivalents) resulted in moderate cavitation in one animal and minimal erosion/ulcer with secondary epidermal hyperplasia at the infusion site in another animal. Based on the data from the previous dog tolerability study, the doses used in the study described in this report were anticipated to be well tolerated and achieve measurable systemic exposure of TFV in dog plasma.

## RESULTS

### Dose formulation analyses.

All dose formulation solution stability samples were stable at room temperature for up to seven days and fourteen days at 2 to 8°C. During the study, the results of all dose formulations samples were found to be within the acceptance criteria, except for the 8.33 μg/ml formulation (group 2) samples collected on day 1 for which concentrations of 86.8 and 87.3% of the theoretical concentration were recorded in the rat study. The impact on the study was determined to be minimal, given that the incorrect formulation was only slightly outside the acceptance window and was only administered for 2 to 5 days of the total 28-day infusion duration. The results of all compatibility samples were found to be within the acceptance criteria, with the exception of one of the 278 μg/ml infusions in the rat study, for which the mean concentration was 88.2% of theoretical after 28 days. Because all other replicate infusions at the same concentration were within the acceptance criteria, the 278-μg/ml formulation was considered compatible with the infusion system.

### In-life observations. (i) Rats.

No TAF-related clinical observations were noted. In addition, no TAF-related effects were observed for body weight, food consumption, ophthalmology examinations, functional observation battery parameters, and respiratory function measurements. No meaningful changes in biomarkers of bone turnover, hormones, or femur pQCT parameters were observed in any group. No changes in organ weight were observed in any group. While observations of erythema and/or edema were recorded for most animals at some point during the study, the incidence and/or severity in the observations were similar when comparing TAF-treated animals to controls and were considered to be due to the presence of the catheter.

### (ii) Dogs.

During the dosing period, in-life observations consisted of discharge at the infusion site with or without the presence of a skin lesion; the presence of a lesion, abscess, or scab at the infusion site; swelling; and warmth and/or discoloration of the infusion site. The affected animals presented with an abnormal gait, hunched posture, and wide stance and appeared to react when the infusion site was palpated, suggesting that the infusion site reactions were associated with significant pain and discomfort. Although infusion site reactions were observed in animals from all treatment groups, including vehicle controls, and were considered a consequence of the continuous s.c. infusion model, the frequency and severity of the reactions and associated observations were greater in TAF-treated animals, suggesting that infusion site reactions were exacerbated by administration of TAF HF.

Other clinical findings that were noted during the dosing phase included elevated body temperature, decreased activity, occasional tremors, dehydration, discolored gums, and discolored pinna. Most of these observations were present across all treatment groups, including vehicle controls, and were likely associated with the inflammation at the infusion site and the overall deteriorating physical condition of the animals.

TAF-related effects on erythema and edema were observed at doses of ≥25 μg/kg/day for females; a slight increase in incidence of animals observed with very slight to moderate edema and very slight to severe erythema was observed at doses of ≥25 μg/kg/day compared to controls throughout the study. There were no TAF-related effects on erythema or edema for males; no edema was observed, and either no erythema or very slight erythema (barely perceptible) was recorded.

There were no TAF-related changes in body weight, food consumption, ophthalmology and electrocardiology evaluations, urinalysis and urine chemistry parameters, biomarkers of bone turnover (bone-specific alkaline phosphatase [BAP] and C-terminal telopeptides of type I collagen [CTx]), hormones (parathyroid hormone 1-84 (PTH), 1,25-dihydroxyvitamin D, bone densitometry parameters (distal radius metaphysis and distal radius diaphysis), or organ weight changes.

### Mortality. (i) Rats.

There were three preterminal deaths in the recovery group occurring between days 10 and 26 of the dosing phase. These preterminal deaths included one female administered the vehicle, one female administered 100 μg/kg/day, and one female administered 300 μg/kg/day. In all cases, euthanasia was performed as a result of clinical findings related to the infusion site. Hematology and biochemistry changes in these animals were generally similar to those for terminally euthanized animals and confirmed the presence of an inflammatory process. Other observations in these animals included mild to moderate s.c. inflammation (mixed and/or mononuclear) and moderate to marked ulceration of the skin at the infusion site with bacterial infection noted. Draining lymph nodes (axillary and/or inguinal) were noted to be enlarged macroscopically in all three females, which correlated with plasmacytosis microscopically. All three females also had minimal to mild increased cellularity (myeloid) within the bone marrow. In addition, in one female, the spleen was noted to be enlarged macroscopically, which correlated with mildly increased extramedullary hematopoiesis microscopically. These findings in the draining lymph nodes, bone marrow, and spleen were considered to be secondary and reactive to the inflammation at the infusion site. Based on the clinical pathology and microscopic findings, the cause of preterminal euthanasia in these females was most likely due to complications from the procedure (s.c. infusion) and opportunistic infection of the infusion site by bacteria and therefore not due to the administration of TAF.

In addition to the preterminal euthanasia that occurred in the recovery animals, there were two animals from the PBMC subset of animals that were either found dead or euthanized preterminally. In both cases, the cause of morbidity could not be determined. On day 16 of the dosing period, one female administered 1,000 μg/kg/day had a swollen surgical site with the presence of skin scab, dryness, and discoloration. The animal was euthanized due to suspicion of necrosis in the tissue surrounding the surgical site (lumbar); however, no macroscopic findings were observed at necropsy. On day 31 (day 3 of the recovery period), one male administered 300 μg/kg/day was found dead in his cage. No abnormal observations had been recorded previously for this animal, and there were no abnormal findings at necropsy. Since both of these animals were part of the PBMC subset, histologic evaluation was not performed as per the study plan.

### (ii) Dogs.

In total, there were 16 unscheduled euthanasia, which occurred between days 5 and 20. Unscheduled euthanasia included two males administered vehicle (days 5 and 18), one male from the 25-μg/kg/day dose group (day 13), one male from the 83.3-μg/kg/day dose group (day 5), and one male from the 833-μg/kg/day dose group (days 5). All remaining animals administered TAF at a dose of 833 μg/kg/day underwent unscheduled euthanasia on day 12 or 13 for the males and on day 19 or 20 for the females. All 16 unscheduled euthanasia were a result of significant swelling and/or discharge at the infusion site and deteriorating physical condition. The gross findings noted at the infusion site of these dogs were considered to be complications of the experimental procedure (continuous s.c. infusion) since similar changes were observed in the control animals and at doses of ≤83.3 μg/kg/day. The frequency and severity of the gross findings were higher in the 833-μg/kg/day dose group, suggesting that the infusion site reactions were exacerbated by treatment with the high dose of TAF in this group. Microscopic findings, including TAF-related findings, were similar to those observed at scheduled euthanasia on day 29. Specific details will be discussed in the gross pathology and histopathology results below.

### Hematology. (i) Rats.

Administration of TAF HF to rats elicited changes in hematology parameters at a dose of ≥30 μg/kg/day. These changes included increases in white blood cell counts (WBC), neutrophil counts (NEUT), lymphocyte counts (LYMPH), monocyte counts (MONO), eosinophil counts (EOS), and platelet counts (PLT).

On day 14 of the dosing period, hematology changes included increases in NEUT (+42% to +177%) and MONO (+47% to +261%) in males and females at doses of ≥30 μg/kg/day. These changes were minimal to mild and, although not dose dependent, were considered to be related to the administration of TAF. At the end of the dosing period on day 29, TAF-related hematology changes consisted of increases in NEUT (≥100 μg/kg/day; +39% to +105%) and MONO (≥30 μg/kg/day; +61% to +88%), as well as increases in LYMPH (1,000 μg/kg/day; +43% to +53%) and EOS (≥30 μg/kg/day; +97% to +221%) in males and females. These changes reflected increases in WBC count observed in most of the affected groups (+29% to +82%). There was also a minimal increase in PLT in males administered 1,000 μg/kg/day on day 29 (+25%). Increases in leucocytes (WBC, MONO, NEUT, LYMPH, and EOS) during the dosing period were indicative of an inflammatory process and were associated with swollen and oozing infusion sites (tissue fluid consisting of a mixture of blood/leucocytes and lymph fluid), as well as microscopic mixed and/or mononuclear cell inflammation, necrosis, edema, fibrosis, and hemorrhage in most animals. The lack of a clear dose-response in these parameters on day 14 or 29 may have been associated with administration of nonsteroidal anti-inflammatory (meloxicam), opioid (buprenorphine), and/or antibiotic (TMS) treatments throughout the dosing period. At the end of the recovery period (day 57), increases in MONO (+54% to +96%), LYMPH (+64%), and WBC (+42% to +59%) counts were still observed for males and/or females at all doses, indicative of a residual inflammatory process.

### (ii) Dogs.

Administration of TAF to dogs elicited changes in hematology parameters at doses of 25, 83.3, and 250 μg/kg/day. These changes included minimal to mild increases in WBC counts (+28% to 71%), NEUT counts (+37% to 92%), and MONO counts (+55% to 113%) on day 14 and/or day 28 in some individuals administered TAF at doses of ≥25 μg/kg/day. Although the changes in these parameters were considered TAF related, they did not appear to be dose dependent. The lack of a clear dose-response may be due, in part, to the administration of nonsteroidal anti-inflammatory (meloxicam and caprofen), opioid (buprenorphine), and/or antibiotic (amoxicillin-clavulanate and cephalexin) treatments throughout the dosing period based on need. Minimal decreases in red cell mass (red blood cell, hemoglobin, and hematocrit) were observed in a few individual males and females that received TAF at doses of ≥25 μg/kg/day; however, the observed changes were of lower or comparable magnitude compared to the control group. As a result, the changes in red cell mass were considered to be procedural in origin and reflective of blood loss secondary to multiple blood collections and/or inflammation and hemorrhage at the infusion site. All changes in hematology parameters appeared to be reversible since the observed changes returned to normal at the end of the recovery period (day 56).

### Coagulation. (i) Rats.

Administration of TAF HF to rats was associated with changes in coagulation parameters at ≥30 μg/kg/day. There was a minimal TAF-related increase in fibrinogen (FIB) concentration in males (+2 to +60%) and females (+2% to 21%) on day 14 and/or 29 at doses of ≥30 μg/kg/day. FIB concentrations returned to normal at the end of the recovery period; therefore, the increases were considered to be reversible.

### (ii) Dogs.

Administration of TAF to dogs was associated with changes in coagulation parameters at the 83.3- and 250-μg/kg/day doses. These changes included a minimal to mild increase in FIB (+45% to 85%) observed on day 14 and/or day 28 in some individuals administered TAF at doses of 83.3 and 250 μg/kg/day. Although considered to be TAF related, the increase in FIB, which was associated with increases in NEUT and/or MONO, was generally consistent with an inflammatory process and did not appear to be dose dependent. The lack of a clear dose-response in FIB increase may have been secondary to the veterinary treatments (anti-inflammatory and/or antibiotics) administered throughout the study and that may have attenuated the acute-phase response. The increase in FIB was considered reversible since the change appeared to fully resolve at the end of the recovery period (day 56).

### Clinical chemistry. (i) Rats.

Administration of TAF HF to rats was associated with minimal changes in clinical chemistry parameters at doses of ≥30 μg/kg/day. Alterations occurred in alkaline phosphatase activity (ALP), glucose (GLUC), total protein (TPROT), albumin (ALB), globulin (GLOB), albumin/globulin ratio (A/G ratio), and urea nitrogen (UREAN) concentrations. Minimal increases in ALP in females were observed at doses of ≥100 μg/kg/day on days 14 and/or 29 (+38% to +117%). In addition, minimal to mild increases in GLUC were observed in males at doses of ≥100 μg/kg/day on days 14 and/or 29 (+17% to +50%). Increases in UREAN concentrations were also noted in females at doses of ≥30 μg/kg/day on day 29 (+16% to +24%) and in males at doses of ≥100 μg/kg/day on day 29 (+15% to +29%). Mild decreases in ALB concentration in females at doses of ≥30 μg/kg/day (−13% to −17%) and in males at doses of ≥300 μg/kg/day (−9% to −16%) were observed on days 14 and/or 29 and minimal to mild increases in GLOB concentration were observed in females at doses of ≥30 μg/kg/day on day 14 (+6% to +15%) and in males at doses of ≥30 μg/kg/day on days 14 and/or 29 (+8% to +32%). These changes in proteins fractions were reflected in the A/G ratio, which decreased in males and females at doses of ≥30 μg/kg/day on days 14 and/or 29 (−9% to −37%), and in the TPROT concentration, which decreased in females at doses of ≥30 μg/kg/day on day 14 (−5% to −8%).

The lack of a clear dose-response in clinical chemistry parameters on days 14 and 29 may have been associated with administration of nonsteroidal anti-inflammatory (meloxicam), opioid (buprenorphine), and antibiotic (TMS) treatments throughout the dosing period.

At the end of the recovery period, changes in ALP in females (1,000 μg/kg/day; +49%), UREAN concentration in males (≥300 μg/kg/day; +13% to 22%), GLOB concentration in males (1,000 μg/kg/day; +13%), in A/G ratio in males (≥30 μg/kg/day; −10% to −21%) and females (≥300 μg/kg/day; −10% to −12%) were still evident, although at a generally lower magnitude, indicating partial reversibility.

### (ii) Dogs.

Administration of TAF to dogs was associated with changes in clinical chemistry parameters at doses of ≥25 μg/kg/day. These changes included minimal to mild decreases in ALB concentration (−2% to −16%), increases in GLOB concentration (+10% to 20%), and subsequent decreases in the A/G ratio (−11% to −22%). These changes were observed on day 14 and/or day 28 in some individuals administered TAF at doses of 25, 83.3, or 833 μg/kg/day. Although these changes were considered to be TAF related, they were not dose dependent. The lack of a clear dose-response in the ALB, GLOB, and A/G changes may have been secondary to the veterinary treatments (anti-inflammatory and/or antibiotics) administered throughout the study, which may have attenuated the acute-phase response. The TAF-related changes in clinical chemistry were considered reversible since the described changes had resolved at the end of the recovery period (day 56).

Minimal to mild increases in alkaline phosphatase activity were observed in a few males and females in the 25-, 83.3-, or 250-μg/kg/day dose groups during the main study. The observed increases in alkaline phosphatase activity were inconsistent, transient in nature, independent of dose, and low in incidence; therefore, the changes were not considered to be related to the administration of TAF HF but ascribed to possible stress related to the procedure and/or inflammation at the infusion site.

### Gross pathology. (i) Rats.

Notable gross pathology findings are summarized in Table 1. Overall, there were no TAF-related macroscopic findings on day 29 (terminal euthanasia animals) or day 57 (recovery euthanasia animals). On day 29, all males receiving TAF at doses of 1,000 μg/kg/day were noted to have masses macroscopically at the infusion and/or the catheter exteriorization site, which generally correlated with mild to marked, mixed and/or mononuclear inflammation, with various degrees of fibrosis, necrosis, and/or edema microscopically. The presence of bacteria was confirmed in all males from this group and in females from both the control and treatment groups. The apparent dose-related increased incidence and severity of mixed cell inflammation with bacteria observed at the infusion site in males at the end of the main study was morphologically similar to what was observed in three of five control females. Therefore, the mixed cell inflammation with bacterial infection was considered secondary to the skin ulceration (opportunistic infection) resulting from the experimental procedure and not directly due to the administration of TAF HF.

**TABLE 1:** Summary of notable gross pathology findings in rats: scheduled euthanasia (day 29)

Parameter	Gender and group									
	Males					Females				
	1	2	3	4	5	1	2	3	4	5
Dose (μg/kg/day)	0	30	100	300	1,000	0	30	100	300	1,000
No. of animals/group	5	5	5	5	5	5	5	5	5	5
										
Site, infusion (no. examined)	5	5	5	5	5	5	5	5	5	5
Mass	0	1	1	1	4	1	0	0	0	1
Swelling	0	1	0	0	0	2	0	0	0	1
Thick	0	2	1	2	0	2	2	3	3	3
Focus, dark	0	2	0	1	1	2	0	1	1	0
Nodule	0	0	0	0	1	0	0	0	0	0
Material accumulation, pale	0	0	0	0	0	0	1	2	0	2
										
Catheter, exteriorization site (no. examined)	1	1	1	0	2	2	4	4	4	3
Abrasion, dark	0	0	1		0	0	0	2	0	1
Mass	0	0	0		1	0	2	1	0	0
Focus, dark	0	0	0		0	1	0	0	0	0
Thick	1	0	0		2	1	3	1	3	2
Swelling	0	1	0		0	2	3	4	3	2
Scab, dark	0	0	0		2	0	0	1	2	2
Nodule	0	0	0		1	0	0	0	0	0

At the end of the recovery period (day 57), masses were observed macroscopically in three females that had received TAF at a dose of 1,000 μg/kg/day, which correlated to microscopic findings of inflammation (mononuclear and/or mixed cell) with fibrosis, edema and/or necrosis, as well as bacterial infection. In addition, a skin ulceration was observed in one of these animals. Bacteria were not noted in any females that did not have a mass macroscopically.

### (ii) Dogs.

Gross findings noted at the infusion site and subcutis at the end of the dosing period (day 29) or at unscheduled euthanasia included increases in mass thickness and, swelling coupled with fluid accumulation. The incidence of these findings was similar between control and treated animals and no clear pattern was observed; however, compared to controls, a variable, dose-independent increase in the severity was noted in some terminal animals administered TAF at doses of ≥25 μg/kg/day. Therefore, the gross findings were considered complications of the experimental procedure (continuous s.c. infusion), with probable TAF-associated exacerbation. Other gross findings observed at the end of the main study (day 29) were considered unrelated to the administration of TAF HF due to the fact that they were considered incidental, of the nature commonly observed in this strain and age of dogs, and/or of similar incidence in control.

Gross findings noted at the infusion site at the end of the recovery study (day 57) included increases in mass, thickness, and swelling. The incidence of these findings was similar between control and treated animals, and/or no suggestive patterns such as dose-related changes were noted. Furthermore, the severity of the infusion site grading in each group at the end of the recovery period (day 57) was lower than at the end of the dosing period (day 29). Therefore, the gross findings were considered evidence of ongoing resolution of TAF-related exacerbation of the experimental procedure complications (continuous s.c. infusion). Other gross findings observed at the end of the recovery period (day 57) were considered unrelated to administration of TAF HF due to the fact they were considered incidental, of the nature commonly observed in this strain and age of dogs, and/or of similar incidence in control and treated animals.

### Histopathology. (i) Rats.

TAF-related microscopic findings were limited to the infusion site of males at the end of the treatment phase (day 29) as summarized in Table 2. Microscopic examination revealed a dose-related increased incidence and severity of fibrosis and mononuclear cell inflammation at doses of ≥300 μg/kg/day and necrosis in the 1,000-μg/kg/day dose group at the infusion site in males only.

**TABLE 2:** Summary of noteworthy microscopic findings in rats: scheduled euthanasia (day 29)

Parameter	Gender and group^*a*^									
	Males					Females				
	1	2	3	4	5	1	2	3	4	5
Dose (μg/kg/day)	0	30	100	300	1,000	0	30	100	300	1,000
No. of animals/group	5	5	5	5	5	5	5	5	5	5
										
Site, infusion (no. examined)	5	5	5	5	5	5	5	5	5	5
Fibrosis	(5)	(5)	(5)	(5)	(5)	(5)	(5)	(5)	(5)	(5)
Minimal	4	0	2	0	0	1	0	0	0	0
Mild	0	2	1	2	1	3	3	3	3	2
Moderate	1	2	2	3	3	1	2	2	2	3
Marked	0	1	0	0	1	0	0	0	0	0
Inflammation, mixed cell	(1)	(4)	(5)	(3)	(5)	(3)	(5)	(5)	(4)	(5)
Minimal	1	2	2	0	0	1	1	1	0	0
Mild	0	0	1	3	1	0	0	2	2	4
Moderate	0	2	2	0	1	1	3	2	2	1
Marked	0	0	0	0	3	1	1	0	0	0
Inflammation, mononuclear cell	(5)	(5)	(5)	(5)	(4)	(5)	(5)	(5)	(5)	(4)
Minimal	4	1	3	1	0	5	1	4	2	1
Mild	1	3	2	2	0	0	4	1	3	3
Moderate	0	1	0	2	4	0	0	0	0	0
Necrosis	(3)	(2)	(1)	(1)	(5)	(1)	(1)	(2)	(0)	(1)
Minimal	3	2	1	1	1	1	1	2	0	1
Mild	0	0	0	0	4	0	0	0	0	0
Ulceration, skin	(2)	(1)	(1)	(2)	(2)	(1)	(2)	(3)	(2)	(4)
Minimal	0	1	1	1	1	0	1	0	1	1
Mild	1	0	0	1	0	0	0	1	0	2
Moderate	1	0	0	0	1	1	0	2	1	0
Marked	0	0	0	0	0	0	1	0	0	1
										
Site, infusion; BB^*b*^ (no. examined)	5	5	5	5	5	5	5	5	5	5
Bacteria present	1	2	4	3	5	3	4	5	4	5

a Numbers in parentheses represent the number of animals with the finding.

b BB, Brown and Brenn staining.

Mixed cell inflammation with bacterial infection (confirmed microscopically and by Brown and Brenn staining) at the infusion site was frequently observed at the skin surface in conjunction with ulcerated skin lesions (which was observed microscopically at the infusion site in males and females in both the control and TAF HF treatment groups). In general, bacteria were found in almost all animals with mixed cell inflammation at the infusion site. Although the incidence of this finding was lower in control males, the incidence and severity of mixed cell inflammation in control females was comparable to that observed in other groups, including males (Table 2). This mixed cell inflammation with an opportunistic bacterial inflammation was considered secondary to the ulcerated skin lesions associated with the experimental procedure and not directly due to the administration of TAF HF.

Microscopic findings noted at the infusion site were still observed at the end of the recovery period on day 57. The TAF-related findings noted at the infusion site (increased incidence and severity of fibrosis, mononuclear cell inflammation and necrosis) were generally of reduced incidence and/or severity compared to the main study animals. This suggests ongoing resolution of TAF-related exacerbation of infusion site lesions noted in males at the end of the main phase.

### (ii) Dogs.

TAF-related microscopic findings on day 29 (scheduled euthanasia) included necrosis, mononuclear cells inflammation and mixed cells inflammation at the infusion site (Table 3). At the end of the dosing period, an increased incidence/severity of mononuclear cell inflammation present in the fibrotic capsule of the infusion site was noted at TAF doses of ≥25 μg/kg/day. An increased incidence of mixed cell inflammation was also noted in the dermis/subcutis surrounding and often disrupting the fibrotic capsule at the infusion site at TAF doses of ≥25 μg/kg/day. Minimal to mild necrosis characterized by tissue pallor, cellular debris, and pyknotic cells around the infusion site/cavity was noted in males receiving TAF doses of ≥25 μg/kg/day and in females receiving TAF doses of ≥83.3 μg/kg/day. In addition, vascular necrosis was noted at the vicinity of the infusion site in one male receiving a TAF dose of 250 μg/kg/day. For unscheduled euthanasia animals, the mononuclear cell inflammation and necrosis noted at the infusion site was generally more severe compared to animals terminally euthanized at the end of the dosing period; however, the severity of mixed cells inflammation was not meaningfully different from that observed in the main study. Minimal and focal necrosis was noted in only one animal, which was a male from the control group that had undergone unscheduled euthanasia. Rare (low in number) Gram-positive bacteria were observed at the infusion sites of seven terminal/unscheduled animals with the Brown and Brenn stain, four of which received antibiotic treatment during the course of the study. However, the localization/appearance of the bacteria in some of these animals may have been artefactual (sampling and/or processing). The remaining microscopic findings noted at the infusion site (fibrosis, edema, and hemorrhages) were of similar incidence and severity between groups, with no suggestive pattern, and were considered related to the experimental procedure. Other microscopic findings observed at the end of the main study, including increased myeloid cellularity of the bone marrow and decreased lymphoid cellularity in the thymus, were considered incidental, related to the experimental procedure (s.c. infusion and stress related changes), of the nature commonly observed in this strain and age of dogs, and/or were of similar incidence and severity in control and treated animals and therefore were considered unrelated to administration of TAF HF.

**TABLE 3:** Summary of microscopic findings in dogs: scheduled euthanasia (day 29)

Parameter	Gender and group^*a*^							
	Males				Females			
	1	2	3	4	1	2	3	4
Dose (μg/kg/day)	0	25	83.3	250	0	25	83.3	250
No. of animals/group	2	3	3	4	4	4	4	4
Site, infusion (no. examined)	2	3	3	4	4	4	4	4
								
Inflammation, mononuclear cell	(2)	(3)	(3)	(4)	(2)	(4)	(4)	(4)
Minimal	2	0	0	0	0	2	0	0
Mild	0	1	0	0	1	0	1	0
Moderate	0	2	3	2	1	1	1	3
Marked	0	0	0	2	0	1	2	1
								
Necrosis	(0)	(2)	(2)	(3)	(0)	(0)	(1)	(1)
Minimal	0	1	2	1	0	0	1	1
Mild	0	1	0	2	0	0	0	0
								
Inflammation, mixed cell	(1)	(3)	(2)	(3)	(2)	(4)	(3)	(3)
Minimal	0	0	2	1	0	3	2	1
Mild	0	1	0	1	0	1	1	0
Moderate	0	0	0	0	2	0	0	0
Marked	1	1	0	0	0	0	0	2
Severe	0	1	0	1	0	0	0	0

a Numbers in parentheses represent the numbers of animals with the finding.

Microscopic findings noted at the scheduled euthanasia of the dosing period were also observed at the end of the recovery study euthanasia (day 57) and are summarized in Table 4. An increased incidence/severity of TAF-related mononuclear cells inflammation in the fibrotic capsule of the infusion was noted in males and females at doses of ≥25 μg/kg/day at the end of recovery study (day 57). An increased incidence/severity of mixed cell inflammation in the dermis/subcutis surrounding and often disrupting the fibrotic capsule of the infusion site was also noted at doses of ≥25 μg/kg/day. However, the severity and/or incidence of both findings were lower compared to the end of the dosing period (day 29) and were therefore interpreted as evidence of ongoing resolution of the TAF-related microscopic findings. Rare (low in number) Gram-positive bacteria were observed at the infusion site of two recovery animals. The presence of bacteria correlated with the presence of mixed cell inflammation in those two animals. The two animals did not receive antibiotics during the course of the study. Other microscopic findings observed at the end of the recovery study were considered incidental, associated with the experimental procedure (s.c. infusion), of the nature commonly observed in this strain and age of dogs, and/or were of similar incidence and severity in control and treated animals and therefore were considered unrelated to the administration of TAF HF.

**TABLE 4:** Summary of microscopic findings in dogs: scheduled euthanasia (day 57)

Parameter	Gender and group^*a*^							
	Males				Females			
	1	2	3	4	1	2	3	4
Dose (μg/kg/day)	0	25	83.3	250	0	25	83.3	250
No. of animals/group	2	2	2	2	2	2	2	2
Site, infusion (no. examined)	2	2	2	2	2	2	2	2
								
Inflammation, mononuclear cell	(0)	(1)	(1)	(2)	(1)	(1)	(1)	(2)
Minimal	0	1	0	1	1	0	0	2
Mild	0	0	1	0	0	0	0	0
Moderate	0	0	0	1	0	1	1	0
								
Inflammation, mixed cell	(0)	(0)	(0)	(2)	(0)	(1)	(1)	(0)
Minimal	0	0	0	0	0	0	0	0
Mild	0	0	0	1	0	0	0	0
Moderate	0	0	0	1	0	1	0	0
Marked	0	0	0	0	0	0	1	0

a Numbers in parentheses represent the numbers of animals with the finding.

### Toxicokinetics. (i) Rat plasma.

The concentrations of TAF in rat plasma were BLQ in nearly all samples from all dose groups; therefore, TAF TK parameters could not be estimated. TK parameters estimated for TFV are presented in Table 5. No consistent sex-related differences in TFV TK were observed. Based on combined data from males and females, steady-state was achieved within the first 48 h of the start of infusion in all dose groups and was maintained throughout the infusion period. The estimated *C*_ss_ values for TFV were 0.428, 1.62, 4.86, and 17.4 ng/ml in the 30-, 100-, 300-, and 1,000-μg/kg/day dose groups, respectively, indicating a dose-proportional increase in systemic exposure. Similar dose-proportional increases were observed for *C*_max_ and AUC_last_, with a 33.3-fold increase in dose resulting in a 29.0- and 32.3-fold increase in *C*_max_ and AUC_last_, respectively. Plasma concentrations of TFV declined rapidly at the end of treatment, with all samples returning to BLQ between 24 and 168 h post-end of infusion in all dose groups.

**TABLE 5:** Composite plasma TFV TK parameters in male and female rats following continuous s.c. infusion of TAF^*a*^

TAF dose (μg/kg/day)	Sex	*T*_max_ (h)	*C*_max_ (ng/ml)	*C*_ss_ (ng/ml)	TimeC_ss_ (h)	AUC_last_ (h · ng/ml)
30	M	336	0.680	0.504	6.77	389
	F	504	0.676	0.400	0.895	352
	M+F	504	0.658	0.428	0.925	371
100	M	672	1.85	1.49	15.7	1,070
	F	504	3.10	1.80	40.3	1,340
	M+F	504	2.43	1.62	20.5	1,210
300	M	48	7.44	5.46	49.9	3,480
	F	504	5.78	4.14	20.1	2,900
	M+F	168	6.14	4.86	42.4	3,190
1,000	M	504	24.1	19.9	41.9	13,800
	F	96	17.7	14.9	6.11	10,200
	M+F	168	19.1	17.4	21.2	12,000

a M, male; F, female; M+F, combined male and female; TimeC_ss_, time to steady-state concentration.

### (ii) Dog plasma.

The TK parameters estimated for TAF are presented in Table 6. TAF was undetectable in nearly all samples from control animals. No consistent sex-related differences in TAF TK were observed. Based on combined data from males and females, steady-state was achieved within the first 4 h after the start of the infusion in all dose groups and was maintained throughout the infusion period. The estimated *C*_ss_ values for TAF were 0.0967, 0.389, 1.53, and 5.97 ng/ml in the 25-, 83.3-, 250-, and 833-μg/kg/day dose groups, respectively, suggesting that the systemic exposure increased slightly greater than dose proportionally. The plasma concentrations of TAF decreased rapidly at the end of treatment, with TAF levels falling to below the LLOQ by 24 h post-end of infusion.

**TABLE 6:** Mean plasma TAF TK parameters in male and female dogs following continuous s.c. infusion of TAF^*a*^

TAF dose (μg/kg/day)	Sex	*n*	Mean (SD)^*b*^					
			*T*_max_ (h)	*C*_max_ (ng/ml)	*C*_ss_ (ng/ml)	TimeC_ss_ (h)	*t*_1/2_ (h)	AUC_672_ (h · ng/ml)
25	M	6	132 [2–504]	0.188 (0.056)	0.117 (0.028)	0.5 [0.4–0.6]	ND	90.5 (19.3)^1^
	F	6	96 [12–336]	0.183 (0.101)	0.0764 (0.0590)	0.3 [0.1–0.6]	ND	74.2 (30.9)^2^
	M+F	12	96 [2–504]	0.185 (0.078)	0.0967 (0.049)	0.5 [0.1–0.6]	ND	84.4 (23.6)^4^
83.3	M	6	192 [12–672]	0.645 (0.155)	0.362 (0.087)	0.8 [0.7–1.8]	ND	280 (69)^1^
	F	6	96 [4–504]	0.863 (0.249)	0.416 (0.166)	1.2 [0.5–4.9]	ND	299 (60)^3^
	M+F	12	96 [4–672]	0.724 (0.214)	0.389 (0.129)	0.9 [0.5–4.2]	ND	288 (62)^5^
250	M	6	36 [1–336]	2.30 (0.56)	1.47 (0.41)	1.5 [1.0–35]	ND	829 (296)
	F	6	132 [8–336]	2.61 (0.61)	1.59 (0.29)	7.9 [1.0–91]	ND	923 (196)
	M+F	12	72 [1–336]	2.45 (0.58)	1.53 (0.34)	3.5 [1.0–91]	ND	876 (244)
833	M	6	48 [2–96]	7.62 (2.94)	5.61 (2.51)	1.6 [0.9–29]	ND	ND
	F	6	36 [2–96]	9.12 (2.23)	6.33 (1.83)	2.7 [9.4–156]	ND	ND
	M+F	12	48 [2–168]	8.37 (2.61)	5.97 (2.13)	2.5 [0.9–156]	ND	ND

a M, male; F, female; M+F, combined male and female.

b Data for *T*_max_ and TimeCss are reported as “medians [ranges].” ND, no data. Superscript numbers: 1, *n* = 5; 2, *n* = 3; 3, *n* = 4; 4, *n* = 8; and 5, *n* = 9.

The plasma TK parameters estimated for TFV are presented in Table 7. Similar to TAF, TFV was undetectable in nearly all samples from control animals, and no consistent sex-related differences in TFV TK were observed. Based on combined data from males and females, the median time to steady-state in all dose groups was ∼100 h post-start of infusion, and steady state was maintained throughout the infusion period. The estimated *C*_ss_ values for TFV were 1.26 ± 0.40, 4.39 ± 1.09, 13.1 ± 2.56, and 34.6 ± 15.9 ng/ml for the 25-, 83.3-, 250-, and 833-μg/kg/day dose groups, respectively, indicating a dose-proportional increase in TFV systemic exposure. Similar dose-proportional increases were observed for *C*_max_ and AUC_0–672_ between the 25- and 250-μg/kg/day dose groups, with a 10-fold increase in dose resulting in 9.8- and 10.5-fold increases in *C*_max_ and AUC_0–672_, respectively. Because all animals in the 833-μg/kg/day dose group were terminated early, dose proportionality in *C*_max_ and AUC_0–672_ could not be accurately assessed. Plasma concentrations of TFV declined slowly in recovery animals after the cessation of treatment. The terminal half-lives were estimated to be 80.7 ± 13.9, 175 ± 81.5, and 263 ± 60 h in the 25-, 83.3-, and 250-μg/kg/day dose groups, respectively, indicating an apparent increase in half-life with increasing dose. Again, because animals in the 833-μg/kg/day dose group were terminated early, terminal elimination data were not available for these animals.

**TABLE 7:** Mean plasma TFV TK parameters in male and female dogs following continuous s.c. infusion of TAF^*a*^

TAF dose (μg/kg/day)	Sex	*n*	Mean (SD)^*b*^					
			*T*_max_ (h)	*C*_max_ (ng/ml)	*C*_ss_ (ng/ml)	TimeC_ss_ (h)	*t*_1/2_ (h)	AUC_672_ (h · ng/ml)
25	M	6	504 [96–672]	1.66 (0.19)	1.44 (0.17)	129 [96–129]	NR	862 (52)^1^
	F	6	420 [168–672]	1.59 (0.41)	1.04 (0.50)^1^	126 [0.6–646]^1^	NR	682 (238)
	M+F	12	504 [96–672]	1.62 (0.31)	1.26 (0.40)^4^	126 [0.7–646]^4^	80.7 (13.9)^2^	764 (196)^4^
83.3	M	6	672 [96–672]	5.72 (1.28)	4.65 (0.94)	94 [36–147]	NR	3,110 (480)^1^
	F	6	504 [96–672]	5.41 (0.88)	4.13 (1.26)	121 [89–209]	NR	2,560 (760)
	M+F	12	504 [96–672]	5.56 (1.06)	4.39 (1.09)	107 [36–209]	175 (81.5)^3^	2,810 (680)^4^
250	M	6	420 [168–672]	14.8 (1.5)	12.2 (2.3)	123 [82–528]	NR	7,400 (740)
	F	6	168 [96–672]	16.9 (1.8)	14.0 (2.7)	104 [86–254]	NR	8,600 (1,610)
	M+F	12	336 [96–672]	15.9 (1.9)	13.1 (2.6)	119 [82–528]	263 (60)^3^	8,000 (1,350)
833	M	6	96 [24–168]	32.8 (13.4)	30.3 (14.4)	87 [11–132]	ND	ND
	F	6	168 [96–168]	44.5 (14.7)	38.8 (17.5)	125 [57–195]	ND	ND
	M+F	12	168 [24–168]	38.6 (14.7)	34.6 (15.9)	100 [11–195]	ND	ND

a M, male; F, female; M+F, combined male and female.

b Data for *T*_max_ and TimeCss are reported as “medians [ranges].” ND, no data; NR, not reported (*n* ≤ 2). Superscript numbers: 1, *n* = 5; 2, *n* = 3; 3, *n* = 4; and 4, *n* = 11.

### (iii) Rat PBMCs.

The TK parameters for TFV-DP are presented in Table 8. The intracellular concentrations of TFV-DP in PBMCs were insufficient for estimating TK parameters in males and females from the 30-μg/kg/day dose group and in females from the 100-μg/kg/day dose group. Based on the data from the 300- and 1,000-μg/kg/day dose groups, no consistent sex differences were observed in the TFV-DP TK parameters. Using the combined data from males and females, the estimated intracellular *C*_ss_ values of TFV-DP in PMBCs were 302 and 658 nM and the AUC_last_ values were 140,000 and 396,000 nM ⋅ h at the 300- and 1,000-μg/kg/day dose levels, respectively, indicating that intracellular concentrations in PBMCs increased slightly less than dose proportionally between these two dose groups. TFV-DP concentrations were BLQ in all samples from all groups at the end of the recovery period.

**TABLE 8:** Composite intracellular PMBC TFVDP TK parameters in male and female rats following continuous s.c. infusion of TAF^*a*^

TAF dose (μg/kg/day)	Sex	*T*_max_ (h)	*C*_max_ (nM)	*C*_ss_ (nM)	AUC_last_ (h · nM)
30	M	NC	NC	NC	NC
	F	NC	NC	NC	NC
	M+F	NC	NC	NC	NC
100	M	504	216	118	67,400
	F	NC	NC	NC	NC
	M+F	NC	NC	NC	NC
300	M	672	316	260	142,000
	F	672	417	344	138,000
	M+F	672	366	302	140,000
1,000	M	336	1,110	706	388,000
	F	168	874	598	394,000
	M+F	336	940	658	396,000

a NC, not calculated; M, male; F, female; M+F, combined male and female.

### (iv) Dog PBMCs.

The TK parameters derived from intracellular concentrations of TFV-DP measured in PMBCs are summarized in Table 9. TFV-DP was not detected in PBMC lysates collected from control animals. No sex-related differences in TFV-DP TK in PBMCs were observed. Based on combined male and female data, the median time for TFV-DP intracellular concentrations to achieve steady-state ranged from 94 to 203 h across all dose groups. Steady-state conditions were maintained for the duration of the treatment period. The estimated intracellular *C*_ss_ values for TFV-DP in PBMCs were 2,260 ± 890, 6,670 ± 2,890, 18,600 ± 7,500, and 42,200 ± 28,100 nM (452 ± 177, 1,330 ± 580, 3,720 ± 1,490, and 8,440 ± 5,620 fmol/million cells) for the 25-, 83.3-, 250-, and 833-μg/kg/day dose groups, respectively, indicating that intracellular TFV-DP concentrations increased in a dose-proportional manner in PBMCs at doses up to 250 μg/kg/day but increased less than dose-proportionally when the TAF dose was increased to 833 μg/kg/day. Between the 25- and 250-μg/kg/day dose ranges, a 10-fold increase in dose resulted in 8.1- and 8.6-fold increases in *C*_max_ and AUC_672_, respectively, which was consistent with the dose-proportional increase observed in *C*_ss_. Because all animals receiving 833 μg/kg/day were terminated early, dose proportionality for *C*_max_ and AUC_672_ could not be accurately assessed at this dose. After the cessation of treatment, the intracellular concentrations of TFV-DP in PBMCs decreased slowly in the surviving recovery animals. The estimated terminal half-lives were 115 ± 59, 185 (*n* = 2), and 199 ± 58 h in the 25-, 83.3-, and 250-μg/kg/day groups, respectively.

**TABLE 9:** Mean intracellular TFV-DP TK parameters in PBMCs from male and female dogs following continuous s.c. infusion of TAF^*a*^

TAF dose (μg/kg/day)	Sex	*n*	Mean (SD)^*b*^					
			*T*_max_ (h)	*C*_max_ (nM)	*C*_ss_ (nM)	TimeC_ss_ (h)	*t*_1/2_ (h)	AUC_672_ (h · nM)
25	M	6	456 [96–672]	3,280 (990)	2,400 (540)^1^	142 [93–600]^1^	NR	1,450 (220)^1^
	F	6	360 [168–672]	3,210 (1,130)	2,070 (1,270)^3^	145 [71–522]^3^	NR	1,220 (430)
	M+F	12	360 [96–672]	3,240 (1,010)	2,260 (890)^4^	142 [71–600]^4^	115 (59)^2^	1,330 (360)^4^
83.3	M	5	552 [168–672]	16,800 (10,800)	7,920 (2,940)	203 [51–603]	NR	5,780 (1,440)
	F	6	360 [168–672]	13,100 (13,900)	5,110 (2,230)^3^	201 [133–249]^3^	NR	3,680 (1,730)
	M+F	11	552 [168–672]	14,800 (12,100)	6,670 (2,890)^4^	203 [51–603]^4^	185 (*n* = 2)	4,640 (1,880)
250	M	6	360 [168–672]	28,800 (8,300)	20,800 (9,000)	174 [69–388]	NR	12,600 (5,300)
	F	6	168 [96–672]	23,500 (9,200)	16,500 (5,400)	145 [70–269]	NR	10,100 (3,600)
	M+F	12	360 [96–672]	26,100 (8,800)	18,600 (7,500)	156 [69–388]	199 (58)^2^	11,400 (4,500)
833	M	5	96 [72–168]	45,100 (23,700)	36,700 (18,800)	70 [57–200]	ND	ND
	F	6	168 [96–360]	56,700 (37,300)	46,800 (35,300)	105 [71–172]	ND	ND
	M+F	11	96 [72–360]	51,400 (30,900)	42,200 (28,100)	94 [57–200]	ND	ND

a M, male; F, female; M+F, combined male and female.

b NR, not reported (*n* ≤ 2); ND, no data. Data for *T*_max_ and TimeC_ss_ are reported as “medians [ranges].” Superscript numbers: 1, *n* = 5; 2, *n* = 3; 3, *n* = 4; and 4, *n* = 9.

## DISCUSSION

In this 28-day toxicity study in rats, continuous s.c. infusion of TAF HF at doses up to 1,000 μg/kg/day resulted in hematology, coagulation, clinical chemistry, and urinalysis changes generally indicative of an inflammatory process; however, these changes may also be attributed, in part, to third-space loss at the infusion site (extracellular compartment or space in the body where fluid does not normally collect). The lack of a clear dose-response in the hematology and clinical chemistry parameters on days 14 and 29 may have been secondary to administration of nonsteroidal anti-inflammatory (meloxicam), opioid (buprenorphine), and/or antibiotic (TMS) treatments throughout the dosing period that may have partially masked a TAF-related exacerbation of an inflammatory response to the presence of the s.c. catheter. In the s.c. tissue surrounding the infusion site in males, the TAF-related microscopic observations that were noted were consistent with an inflammatory process and consisted of exacerbation of fibrosis and mononuclear cell inflammation at doses of ≥300 μg/kg/day, as well as necrosis at the infusion site at the 1,000-μg/kg/day dose. The absence of similar microscopic observations in female rats may be due to their lower body weight, which would have resulted in a 34% lower absolute daily dose of TAF HF and thus lower local exposure to TAF in the s.c. tissue surrounding the infusion site in each treatment group. Although still present at the end of the recovery period, the incidence and/or severity of the observed TAF-related clinical pathology and microscopic changes did diminish, indicating that these changes were trending toward reversibility upon discontinuation of treatment. At the end of the recovery phase (day 57), changes in MONO, LYMPH, and WBC counts were still observed for males and/or females at all doses, indicative of a residual inflammatory process. Changes in ALP activity in females, the UREAN concentration in males (≥300 μg/kg/day), the GLOB concentration in males (1,000 μg/kg/day), the A/G ratio in males (≥30 μg/kg/day) and females (≥300 μg/kg/day), and calcium fractional excretion in females were still observed, although at a generally lower magnitude, indicating partial reversibility. Due to their trend toward reversibility and low severity, the clinical and microscopic observations were not considered adverse. As a result, the systemic and local NOAEL in this study was considered to be 1,000 μg/kg/day, which achieved a high systemic exposure to TFV (*C*_ss_, 17.4 ng/ml; AUC_last_, 120,000 ng ⋅ h/ml) and high intracellular concentrations of TFV-DP in PBMCs (*C*_ss_, 658 nM). In comparison, the NOAEL in a rat toxicity study after 28 days of oral administration of TAF HF was 6.25 mg/kg/day (18), which had the key study finding of dose-related decreases in 1,25(OH)_2_D_3_ (vitamin D). Mechanistic toxicity studies suggest that TAF might directly inhibit vitamin D production, which results in decreased gastrointestinal absorption of calcium and phosphate and decreased renal reabsorption of calcium (19).

In the 28-day toxicity study in dogs, continuous s.c. infusion of TAF HF at doses up to 833 μg/kg/day (TAF free base equivalents) resulted in hematology, coagulation, and clinical chemistry changes that were consistent with an inflammatory response, likely due to a local reaction at the s.c. infusion site. Indeed, macroscopic and microscopic examination of the tissues surrounding the s.c. infusion sites revealed an inflammatory process marked by mononuclear cell inflammation, mixed cell inflammation, and necrosis. For the 833-μg/kg/day dose group, the local inflammation became severe enough that all animals were preterminally euthanized by day 20 due to their deteriorating condition. In animals receiving vehicle or TAF at doses of ≤250 μg/kg/day, the inflammation response was generally less severe, with nearly all animals able to complete the study. In treatment groups receiving TAF at doses of ≤250 μg/kg/day, a clear relationship could not be established between the frequency of observed changes associated with an inflammatory response and the dose of TAF administered, with many changes occurring in vehicle control animals at a similar frequency as in TAF HF-treated animals. Similarly, the severity of the observed changes was often without a clear dose response, although most findings were more severe in TAF HF-treated animals compared to vehicle controls. The lack of a clear dose response in severity may have been due, in part, to the administration of nonsteroidal anti-inflammatory, opioid, and/or antibiotic treatments to animals based on need throughout the dosing period, which could have attenuated the magnitude of the changes associated with the local inflammatory response. Due to the fact that nearly all changes noted were related to a local inflammatory response that was observed in both control and TAF HF-treated animals, the observed changes were considered to be complications of the experimental procedure (continuous s.c. infusion). Because the severity of observations was generally higher in TAF HF-treated animals, treatment with TAF HF was considered to exacerbate the ongoing inflammatory response. Due to the severity of the observations noted, a local NOAEL could not be established in this study. In contrast to the findings related to local inflammation at the infusion site, there was no systemic toxicity associated with exposure to TAF. All preterminal euthanasia, observed clinical signs, clinical pathology changes, and macroscopic and microscopic findings noted were associated with the inflammation observed at the infusion site. As such, the NOAEL for the systemic exposure to TAF, excluding any infusion site-related findings, was considered to be 250 μg/kg/day, which achieved high systemic exposure to both TAF (*C*_ss_, 1.53 ± 0.34 ng/ml; AUC_672_, 876 ± 244 ng ⋅ h/ml) and TFV (*C*_ss_, 13.1 ± 2.6 ng/ml; AUC_672_, 8,000 ± 1,350 ng ⋅ h/ml) and high intracellular concentrations of TFV-DP in PBMCs (*C*_ss_, 18,600 ± 7,500 nM or 3,270 ± 1,500 fmol/million cells). In comparison, the NOAEL in the dog study after 9 months of oral administration of TAF HF was 2 mg/kg/day, which had key study findings of body weight loss, reduction in food consumption, effects on hematology and clinical chemistry parameters, minimal renal toxicity, slightly prolonged PR intervals, dark brown discoloration of the fur (mainly on the extremities), pulmonary findings, macroscopic changes in the kidney, histopathological alterations noted in the kidneys, eyes, lung, spleen, liver, and adrenal glands, decrease in 1,25-dihydroxyvitamin D_3_ (vitamin D), and minimal bone loss at oral doses 3-fold higher than the reported NOAEL.

Based on an analysis performed by Anderson et al. (20) using available data from clinical HIV PrEP studies, the intracellular concentration of TFV-DP in cryopreserved human PBMCs necessary to provide a 90% risk reduction in HIV infection among high-risk populations is ∼200 nM (40 fmol TFV-DP/million cells). Using this target concentration of TFV-DP and fixed-exponent allometric scaling (exponent, 0.75) of TAF, TFV, and TFV-DP PK data from dogs (7) and rabbits (9) after continuous s.c. administration of TAF HF, a reasonable estimate for a clinically relevant dose of TAF for HIV PrEP in humans is ∼150 μg/day (TAF free base equivalents). At this dose, the projected *C*_ss_ of TFV in human plasma and the intracellular *C*_ss_ of TFV-DP in cryopreserved human PMBCs would be 0.200 ng/ml and 200 nM, respectively. Using these clinical dose and exposure estimates, the 1,000-μg/kg/day dose in the rat study achieved a clinical margin of 87.5-fold for TFV, but only 3.3-fold for intracellular TFV-DP, and the 250-μg/kg/day dose in the dog study achieved a clinical margin of 153-fold for TAF, 65.5-fold for TFV, and 93-fold for intracellular TFV-DP.

A foreign body response to the experimental procedure (continuous s.c. infusion) was observed in all dose groups in both rats and dogs, including vehicle, that resulted in significant local inflammation at the site of infusion. The severity of the local inflammation was higher in groups receiving TAF treatment, suggesting that it was exacerbated by administration of TAF HF. According to *Guidance for Industry: Estimating the Maximum Safe Starting Dose in Initial Clinical Trials for Therapeutics in Adult Healthy Volunteers* (21), when therapeutics are administered s.c. and a local toxicity is observed, safety margins for the starting clinical dose should be based on the absolute dose or absolute amount of drug administered, rather than scaling the nonclinical dose to humans using body weight or body surface area. Applying this methodology to the results of this study, the clinical multiples established in female and male rats for the highest dose tested (1,000 μg/kg/day, or 300 and 500 μg/day, respectively) were 2- to 3.1-fold, assuming a daily clinical dose of 150 μg/day. The clinical multiples established in female and male dogs for the lowest dose tested was only 1.2-fold, assuming a daily clinical dose of 150 μg/day. Unfortunately, these low clinical multiples for local TAF HF doses do not support a safe starting dose in initial clinical trials in adult healthy volunteers.

Although TAF HF has been proven to be safe when administered orally (3) and TFV has been safely administered vaginally both as a gel (14, 15) and as an intravaginal ring (16, 17), we have described significant local inflammation following continuous s.c. administration of TAF HF, consisting of increased incidence and severity of fibrosis and mononuclear cell inflammation and necrosis. The TAF-related local inflammation was supported by hematology, coagulation, clinical chemistry and urinalysis changes generally indicative of an inflammatory process. Su et al. (18) have also reported significant inflammation around their s.c. TAF implants which was graded as a severe reaction in both New Zealand White rabbits and rhesus macaque models. The local inflammation occurred even with the lowest TAF dose and flux implant that achieves clinically relevant cellular TFV-DP levels. Other investigators have also reported s.c. tissue pathology at an active TAF implant which delivered locally high drug concentrations along with extended drug exposure (8, 9).

Our main finding in this study is that TAF HF administered continuously as a s.c. infusion appears to exacerbate a background inflammation response to the presence of the s.c. catheter (foreign body response) in the tissue surrounding the infusion site in rats and dogs at all doses tested after 28 days of administration. While the study was confounded by the use of nonsteroidal anti-inflammatory, opioid, and/or antibiotic treatments throughout the dosing period that may have partially suppressed the TAF-related exacerbation of the foreign body response, the severity of the local inflammation was higher in groups receiving TAF treatment compared to vehicle controls. While the findings of local inflammation were considered not adverse in the rat, the occurrence of any local reaction in humans would be unacceptable for an s.c. implant that chronically delivers TAF. Given that an exacerbation of the foreign body response was observed at the lowest dose tested in this study, which delivered a local dose that was less than 10% of the estimated clinically relevant dose, the results from this study suggest that proceeding into clinical trials aimed at investigating s.c. administration of TAF in humans would be met with limited success. It is possible that lower doses and/or lower dose volumes would result in improved tolerability of TAF HF when continuously infused subcutaneously, but those lower doses and/or volumes may result in TFV-DP levels in PBMCs below what is required for effective HIV prevention. Fully discharging the risk of local inflammation and toxicity upon chronic s.c. delivery of TAF in humans will require longer duration nonclinical toxicity studies and an extended clinical study using a s.c. implanted device.

## MATERIALS AND METHODS

### Study design. (i) Rats.

Prior to study initiation, animals were assigned to groups by a stratified randomization scheme designed to achieve similar group mean body weights. The study consisted of five treatment groups (*n* = 35 animals/sex/group), with each group receiving TAF at a dose of either 0, 30, 100, 300, or 1,000 μg/kg/day (equivalent to a local TAF dose of 0, 13.8, 46.0, 138, or 460 μg/day in males or 0, 9.00, 30.0, 90.0, or 300 μg/day in females, based on average body weights of 460 g for males and 300 g for females) (Table 10). Within each treatment group, five animals/sex were assigned to the main study group, and six animals/sex were assigned to the recovery group. Each treatment group also contained two separate satellite groups: one group of six animals/sex for TK and one group of eighteen animals/sex for PBMC sample collection.

**TABLE 10:** Study design for rat toxicity study^*a*^

Group	Test material	Dose level (μg/kg/day)	Dose vol (ml/kg/day)	Dose concn (μg/ml)	Dose rate (ml/kg/h)	No. of animals							
						Main		Recovery		TK		PBMC	
						M	F	M	F	M	F	M	F
1	Vehicle	0	3.6	0	0.150	5	5	5	5	6	6	18	18
2	TAF	30	3.6	8.33	0.150	5	5	5	5	6	6	18	18
3	TAF	100	3.6	27.8	0.150	5	5	5	5	6	6	18	18
4	TAF	300	3.6	83.3	0.150	5	5	5	5	6	6	18	18
5	TAF	1,000	3.6	278	0.150	5	5	5	5	6	6	18	18

a TK, toxicokinetic; PBMC, peripheral blood mononuclear cell; M, male; F, female; TAF, tenofovir alafenamide hemifumarate.

### (ii) Dogs.

Study animals were assigned to groups by a stratified randomization scheme designed to achieve similar group mean body weights. The study consisted of five treatment groups (*n* = 12 animals/sex/group), with each group receiving TAF at a dose of either 0, 25, 83.3, 250, or 833 μg/kg/day (equivalent to a local TAF dose of 0, 180, 600, 1,800, or 6,000 μg/day) (Table 11). Within each treatment group, four animals/sex were assigned to the main study group and two animals/sex were assigned to the recovery group.

**TABLE 11:** Study design for dog toxicity study^*a*^

Group	Test material	Dose level (μg/kg/day)	Dose vol (ml/kg/day)	Dose concn (μg/ml)	Dose rate (ml/kg/h)	No. of animals			
						Main		Recovery	
						M	F	M	F
1	Vehicle	0	3.6	0	0.150	4	4	2	2
2	TAF	25	3.6	6.94	0.150	4	4	2	2
3	TAF	83.3	3.6	23.1	0.150	4	4	2	2
4	TAF	250	3.6	69.4	0.150	4	4	2	2
5	TAF	833	3.6	231	0.150	4	4	2	2

a M, male; F, female; TAF, tenofovir alafenamide hemifumarate.

### (iii) Dog Rx1 and Rx2 dosing period.

Rx1 refers to the first dosing period where male animals were administered the initial formulation in 75 mM citrate buffer (pH 6.0). Rx1 planned to include female animals on a staggered start schedule but based on the initial findings in males; females were not dosed. These Rx1 male animals underwent a second planned surgery during the washout period to change the side of the infusion site before the start of Rx2. Rx2 refers to the second dosing period where both female and male animals were administered the final formulation of 5 mM citrate buffer with 0.8% saline.

### Animals.

The in-life portion of this study was performed at Charles River Laboratories-Montreal (CRL-MTL; Montreal, Quebec, Canada) in compliance with GLP regulations. Care and use of study animals were in compliance with CRL-MTL standard operating procedures (SOPs) under the permission of the Institutional Animal Care and Use Committee.

Naive male and female Sprague-Dawley [Crl:CD(SD)] rats were purchased from Charles River Canada, Inc. (St. Constant, Quebec, Canada). All rats were singly housed in polycarbonate caging with free access to food (Lab Diet Certified CR Rodent Diet 5CR4) and water for the duration of the study, unless otherwise noted. At the time of dose initiation, animals were 11 to 14 weeks old, male rats weighed between 372 and 549 g, and female rats weighed between 252 and 347 g. After an acclimation period of at least 7 days, rats were surgically implanted with a s.c. cannula while under isoflurane anesthesia. Cannulas were routed dorsally through the s.c. tissue extending from the lumbar region to an exteriorization site at the base of the neck. The cannulas were tethered through a swivel system outside the cage, and jackets were placed on the rats to hold the tethered system in place. Saline infusions were maintained at a rate of 0.04 ml/h until study initiation, which occurred after a recovery period of at least 10 days. Antibiotics, opioids, and anti-inflammatory agents were administered during the study at the discretion of veterinary staff in order to maintain animal welfare.

Naive male and female beagle dogs were received from Marshall BioResources (North Rose, NY). Animals were singly housed in stainless steel cages. Animals had free access to water and were offered a ration of food (Lab Diet Certified Canine Diet 5007) once daily for a period of 2 to 4 h, unless otherwise noted. Ambient temperature (17°C to 23°C) and humidity (30 to 70%) were maintained in the study room. The light cycle consisted of 12 h of light followed by 12 h of darkness. At the initiation of Rx1, the males were 8 to 9 months old and weighed between 5.9 and 9.5 kg. At the second initiation (Rx2), animals were 8 to 12 months old, females weighed between 5.7 and 8.4 kg, and males weighed between 6.5 and 9.7 kg. After an acclimation period of at least 7 days, dogs were surgically implanted with a s.c. catheter while under isoflurane anesthesia. Medical grade silicone-based catheters (silastic catheter tubing; Medique, Ville Mont-Royal, Quebec, Canada) were routed dorsally through the s.c. tissue along the left side (females and Rx1 for males) or right side (Rx2 for males) extending from the lumbar region to an exteriorization site at the base of the neck. The catheters were tethered through a swivel system (Lomir Biomedical, Inc., Notre-Dame-de-l’Île-Perrot, Quebec, Canada) outside of the cage and connected to an external infusion pump (Auto Syringe AS50/40; Baxter Healthcare Corporation, Mississauga, Ontario, Canada). After the procedure was complete, jackets (Lomir Biomedical, Inc.) were placed on the dogs to hold the tethered system in place, catheters were filled with saline, and a continuous infusion of saline at a rate of 0.1 ml/h was started. Saline infusions were maintained until study initiation, which occurred after a recovery period of at least 10 days. All infusates were sterile filtered via an in-line 0.22-μm polyvinylidene difluoride (PVDF) filter. Where applicable, any surgical repair of a catheter was performed in accordance with CRL-MTL’s SOPs. Administration of antibiotics and anti-inflammatory medications was performed as recommended by the Study Director and/or Veterinarian when considered appropriate, based on the animal’s health status.

### Test article.

TAF was supplied as the hemifumarate salt by Micro Labs Limited (lot TNO4O17003; Bengaluru, India). The test article appeared as a white to off-white powder and had a purity of 99.7%. All dose calculations were based on the free base form of TAF, which was estimated from the salt conversion factor and lot-specific purity. All concentrations reported for TAF, TFV, and TFV-DP in dose formulations or *in vivo* study samples refer to the free base form.

### Dose formulation and analysis. (i) Rats.

Dosing formulations were prepared two times per week by dissolving the appropriate amount of TAF HF in 5 mM citrate containing 0.8% saline and 0.1% *m*-cresol to afford solutions with TAF concentrations of 0, 8.33, 27.8, 83.3, and 278 μg/ml (TAF free base equivalents). Solutions were sterile filtered, aliquoted into single-use portions, and stored at −80°C. Aliquots were thawed overnight at 4°C prior to use and allowed to warm to room temperature before dosing. Formulations were administered from days 1 to 28 as a continuous s.c. infusion delivered at a rate of 0.150 ml/kg/day using an external syringe pump containing an in-line 0.22-μm PVDF filter. Individual dose rates were calculated based on the most recently recorded body weight.

### (ii) Dogs.

Dosing formulations were prepared two to three times per week by dissolving the appropriate amount of TAF HF in 75 mM citrate buffer (pH 6.0) (Rx1 for males) or 5 mM citrate buffer (pH 6.0) containing 0.8% saline (females and Rx2 for males). Formulations were then sterile filtered through a 0.22-μm PVDF filter, aliquoted into single-use portions, and stored in a freezer set to maintain −80°C. Prior to use, aliquots were thawed overnight in a refrigerator set to maintain 2 to 8°C and then allowed to warm to room temperature for at least 30 min. All dose formulations were infused through an inline 0.22-μm PVDF filter. Individual doses were calculated based on the most recently recorded body weight of each animal.

The stability of TAF in the dose formulations under the conditions encountered during the study was assessed by the Sponsor using a qualified analytical procedure based on HPLC-UV. The concentration of each formulation was measured in duplicate by CRL-MTL upon preparation using a validated analytical procedure based on HPLC-UV. For each formulation, concentration results were considered acceptable when the means of the measured sample concentrations were within or equal to ±10% of the theoretical concentration. Each individual sample concentration result was considered acceptable when it was within or equal to ±15%. An assessment to ensure compatibility of the TAF formulations with the infusion systems used in the study was also performed by CRL-MTL using the same analytical methods and acceptance criteria as the dose formulation analysis.

### In-life observations, measurements, and laboratory evaluations. (i) Rats.

Cage side observations for general health/mortality and moribundity were performed twice daily. Detailed clinical observations and body weight measurements were performed weekly. Food consumption was quantitatively measured weekly starting on day −1 and then daily throughout the dosing and recovery periods, with the exception of the day of scheduled euthanasia. Local irritation assessments for erythema and edema were performed once prior to initiation of dosing and then once weekly until the end of the study; observations were scored according to the modified Draize scoring scale. Ophthalmic examinations were performed once prior to study initiation and again during week 4 of treatment. Functional observation battery (FOB) evaluations were performed on recovery animals once prior to study initiation and on days 14 and 28 and at the end of recovery period. Respiratory function measurement was recorded at least once during the prestudy period, once during week 4 and at the end of recovery. Respiratory measurements were performed on recovery animals for at least 15 min on day 3, once during week 4, and at the end of recovery. Animals were fasted overnight before blood sampling for hematology, coagulation, clinical chemistry, and biochemical markers of bone turnover. Urine was collected from individual animals placed in metabolism cages overnight for urinalysis and urine chemistry.

### (ii) Dogs.

Cage side observations for general health/mortality and moribundity were performed twice daily. The animals were removed from the cage and a detailed clinical observation was performed weekly throughout the study. Individual body weights were recorded weekly, and a fasted body weight was recorded on the day of necropsy. Quantitative food consumption data were recorded daily starting on day −7 and was continued throughout the dosing and recovery periods. Local irritation assessments such as erythema and edema were performed once prior to initiation of dosing and then performed once weekly, until the end of dosing or recovery periods; observations were scored according to the modified Draize scoring scale. Ophthalmic examinations were performed once prior to study initiation and again during week 4 of treatment. Electrocardiology (ECG) evaluations were performed once prior to study initiation, on day 1, and again during week 4 of the treatment period. Animals were fasted overnight before blood sampling for clinical chemistry, hormone measurements, and biochemical markers of bone turnover. Urine was collected overnight from individually housed animals for urinalysis. Densitometry scans and assessments were performed using peripheral quantitative computed tomography (pQCT). Animals were scanned using the same densitometer on each occasion. Bone histomorphometry was performed at 12 days and 5 days prior to terminal euthanasia, using s.c. administered calcein green (8 mg/kg) as a bone labeling agent.

### Toxicokinetic sample collection. (i) Rat plasma.

Blood (∼0.3 ml) was collected via jugular venipuncture from conscious animals in the satellite pharmacokinetic (PK) groups in a sparse sampling design using two subgroups (A and B) per sex per treatment group. Samples from animals in subgroup A (*n* = 3/sex/group) were collected at 0 (predose), 2, 8, 24, 96, 336, and 672 h post-start of infusion and at 24, 336, and 672 h post-end of infusion. Samples from animals in subgroup B (*n* = 3/sex/group) were collected at 1, 4, 12, 48, 168, and 504 h post-start of infusion and at 168 and 504 h post-end of infusion. Blood was collected into tubes containing K_2_EDTA as anticoagulant, separated via centrifugation, and then a 0.100 ml aliquot of plasma was transferred to a tube containing 1 μl of 1 M phenylmethylsulfonyl fluoride (PMSF; esterase inhibitor) in dimethyl sulfoxide (DMSO). The plasma sample was mixed, frozen on dry ice, and stored at −80°C until analysis.

### (ii) Dog plasma.

Blood (∼1.0 ml) was collected via jugular venipuncture from conscious animals (*n* = 4/sex/group) at 0 (predose), 1, 2, 4, 8, 12, 24, 48, 96, 168, 336, 504, and 672 h post-start of infusion. During the recovery period, blood collection occurred at 24, 168, 336, 504, and 672 h post-end of infusion (*n* = 2 animals/sex/group). Blood was collected into tubes containing K_2_EDTA as anticoagulant, separated via centrifugation, and then a 0.35 ml aliquot of plasma was transferred to a tube containing 3.5 μl of 1 M PMSF (esterase inhibitor) in DMSO. The plasma sample was mixed, frozen on dry ice, and stored at −80°C until analysis.

### (iii) Rat PBMCs.

Blood (approximately 10 ml) was collected from the abdominal aorta of anesthetized rats from the satellite PMBC groups using a terminal procedure (*n* = 3 animals/sex/group/time point) at 0 (predose), 168, 336, 504, and 672 h post-start of infusion and at 672 h post-end of infusion. Samples were placed into tubes containing K_2_EDTA and mixed gently, and the PBMCs were isolated using a Ficoll-Paque density method. Each sample of isolated cells was resuspended in 1 ml of phosphate-buffered saline (PBS), and a 20-μl aliquot was removed for determining cell density. The remaining cells were placed in a 1.5-ml conical Eppendorf tube and centrifuged at ambient temperature for 10 min at 300 × *g*. After the supernatant was removed, the resulting pellet was resuspended in 0.5 ml of refrigerated 70% methanol in water. The cell lysates were stored at −80°C until analysis.

### (iv) Dog PBMCs.

Blood (∼10 ml) was collected from conscious animals (*n* = 4/sex/group) via jugular venipuncture at 72, 96, 168, 360, 552, and 672 h post-start of infusion. Due to preterminal euthanasia, in the 833-μg/kg/day dose group, samples for PBMCs were only collected from surviving males through 168 h post-start of infusion and from surviving females through 360 h post-start of infusion. During the recovery period, blood collection occurred at 24, 192, 336, 504, and 672 h post-end of infusion (*n* = 2 animals/sex/group). Samples were collected into tubes containing K_2_EDTA as anticoagulant and mixed gently, and the PMBCs were isolated using a Ficoll-Paque density method. Each sample of isolated cells was resuspended in 1 ml of PBS, and a 20-μl aliquot was removed to determine the cell density. The remaining cells were placed in a 1.5-ml conical Eppendorf tube and centrifuged at ambient temperature for 10 min at 300 × *g*. After the supernatant was removed, the resulting pellet was resuspended in 500 μl of refrigerated 70% methanol in water. The cell lysates were stored at −80°C until analysis.

### Terminal procedures.

Scheduled euthanasia occurred on day 29 (main animals) and day 57 (recovery animals). Euthanized animals were subjected to complete necropsy examination. Images of the infusion site were captured for all animals. Tissue for histopathology examination was collected from the area surrounding the s.c. infusion site and included tissue distal, proximal, and inclusive of the catheter loop, with surrounding skin and muscle.

### Quantification of TAF and TFV in rat and dog plasma.

The concentrations of TAF and TFV in rat plasma were measured using a validated method based on solid-phase extraction followed by liquid chromatography-tandem mass spectrometry (LC-MS/MS) analysis. The method had lower limits of quantification (LLOQs) of 0.010 ng/ml for TAF and 0.100 ng/ml for TFV. Briefly, plasma samples were prepared by adding internal standards (tenofovir alafenamide-d5 and tenofovir-d6) dissolved in 50% methanol in water (vol/vol). The diluted samples were vortex mixed and loaded onto an Oasis MAX 96-well microElution plate (Waters Corporation, Milford, MA). After a series of wash steps, TAF was eluted using 90% (vol/vol) methanol in water, followed by elution of TFV into a separate plate using 0.5% (vol/vol) HCl in methanol. All sample extracts were dried and reconstituted in 40% (vol/vol) methanol in water. Separation was achieved using a 2.1 × 50 mm ACQUITY UPLC HSS T3, 1.8-μm analytical column (Waters Corporation) and a gradient UPLC method utilizing water containing 1% (vol/vol) formic acid (solvent A) and 80% (vol/vol) acetonitrile in methanol (solvent B) as the mobile phase. Detection of analytes and internal standards was performed using a Sciex 6500 triple quadrupole mass spectrometer (Framingham, MA) running an electrospray positive-ion MS/MS method.

### Quantification of TFV-DP in rat and dog PBMC lysates.

The concentration of TFV-DP in rat PBMC lysates was measured at PPD Laboratories (Middleton, WI) using a qualified method. The method was based on solid-phase extraction followed by LC-MS/MS analysis and used dog plasma as a surrogate matrix for constructing calibration standards and quality control samples. The LLOQ was 0.200 ng TFV-DP/ml of PBMC lysate using a 100-μl sample. Briefly, PBMC lysates and surrogate matrix were prepared by adding internal standard (tenofovir diphosphate-d6) dissolved in 70% (vol/vol) methanol in water and then diluting with 20 mM ammonium acetate in water. The diluted samples were vortex-mixed and loaded onto an Oasis WAX 96-well microElution plate (Waters Corporation). After a series of wash steps, TFV-DP was eluted using 2.5% (vol/vol) ammonium hydroxide in methanol. Sample extracts were dried and reconstituted in 60% (vol/vol) methanol in water. Separation was achieved using a 2.1 × 50 mm ThermoScientific BioBasic AX, 5-μm analytical column (Thermo Fisher Scientific, Waltham, MA) and a gradient UPLC method utilizing 5 mM ammonium acetate (pH 6.0) in 70% (vol/vol) acetonitrile in water (solvent A) and 5 mM ammonium acetate (pH 10.5) in 70% (vol/vol) acetonitrile in water (solvent B) as the mobile phase. Detection of TFV-DP and TFV-DP-d6 was performed using a Sciex 6500 triple quadrupole mass spectrometer (Framingham, MA) running an electrospray positive-ion MS/MS method. Concentrations of TFV-DP measured in PBMC lysates were converted to an intracellular concentration using the cell density of each sample prior to lysis and an assumed intracellular volume of 0.2 μl/million cells (7).

### Toxicokinetic analysis.

Toxicokinetic (TK) analysis was performed by Nuventra Pharma Sciences (Durham, NC). For all analytes, estimation of TK parameters, including the maximum concentration (*C*_max_), the time at which each maximum concentration was observed (*T*_max_), and the area under the concentration-time curve from time zero (predose) to the time of the last quantifiable concentration (AUC_last_), were determined based on mean concentration-time data via noncompartmental analysis using Phoenix WinNonlin version 8.1 (Certara USA, Inc., Princeton, NJ). For TFV, the steady-state concentration (*C*_ss_) and the time to reach steady-state (*T_ss_*) were estimated by fitting the mean concentration data to a quadratic plateau regression model (22) using SAS version 9.4 (SAS Institute, Cary, NC). Because of the limited data for TFV-DP, *T*_ss_ could not be estimated, and the *C*_ss_ was estimated as the average of the last three nonzero concentrations measured in each group. All calculations utilized nominal sampling times.
